# Pp65 antigenemia and cytomegalovirus diagnosis in patients with lupus nephritis: report of a series.

**DOI:** 10.1590/2175-8239-JBN-3838

**Published:** 2018-04-26

**Authors:** Katia Lino, Natalia Trizzotti, Fabiana Rabe Carvalho, Rachel Ingrid Cosendey, Cintia Fernandes Souza, Evandro Mendes Klumb, Andrea Alice Silva, Jorge Reis Almeida

**Affiliations:** 1 Universidade Federal Fluminense, Niterói, RJ, Brasil.; 2 Universidade do Estado do Rio de Janeiro, Rio de Janeiro, RJ, Brasil.

**Keywords:** Lupus Erythematosus, Systemic, Cytomegalovirus, Viremia, Lúpus eritematoso sistêmico, Citomegalovírus, Viremia

## Abstract

**Introduction:**

In contrast to organ transplantation, few studies correlate the monitoring of pp65 antigenemia with a diagnosis of cytomegalovirus (CMV) in patients with systemic lupus erythematosus (SLE).

**Objective:**

To highlight the importance of CMV outside transplantation, we monitored pp65 antigenemia in a series of SLE patients.

**Methods:**

From March 2015 to March 2016, SLE patients presenting kidney involvement, fever, and an unclear infection at hospital admission were monitored through pp65 antigenemia. The pp65 antigenemia assay, revealed by immunofluorescence, was correlated with clinical and laboratory findings.

**Results:**

We included 19 patients with a suspected unclear infection. A positivity for pp65 antigenemia was found in seven patients (36.8%). The mean age was 33.5 ± 11.2 years, 16 (84%) were females, and 16 (84%) were black. Lymphopenia, anemia, and higher scores of SLEDAI were significantly more common in pp65-positive patients. Five patients received antiviral therapy with ganciclovir. Although receiving specific CMV treatment, one patient died because of suspected CMV disease.

**Conclusions:**

Pp65 antigenemia might be relevant in SLE patients, and studies with a greater number of patients are needed in order to establish sensitivity and specificity of pp65 antigenemia in different clinical contexts of SLE patients.

## RESUMO

**Introdução:**

Diferentemente do transplante de órgãos, poucos estudos correlacionam o monitoramento da antigenemia pp65 com o diagnóstico de citomegalovírus (CMV) em pacientes com lúpus eritematoso sistêmico (LES).

**Objetivo:**

De modo a destacar a importância do CMV para além do transplante, monitorizamos a antigenemia pp65 em uma série de pacientes com LES.

**Métodos:**

De março de 2015 a março de 2016, pacientes com LES que apresentaram acometimento renal, febre e infecção indeterminada na internação foram monitorados através da antigenemia pp65. O ensaio de antigenemia, revelada por imunofluorescência, foi correlacionado com achado clínicos e laboratoriais.

**Resultados:**

Foram incluídos 19 pacientes com suspeita de infecção indeterminada. Positividade para antigenemia pp65 foi encontrada em sete pacientes (36,8%). A idade média foi de 33,5 ± 11,2 anos; 16 (84%) eram do sexo feminino e 16 (84%) eram negros. Linfopenia, anemia e escore de SLEDAI mais elevado foram significativamente mais comuns em pacientes pp65 positivos. Cinco pacientes receberam terapia antiviral com ganciclovir. Apesar de receber tratamento específico para CMV, um paciente com suspeita de doença por CMV veio a óbito.

**Conclusões:**

Antigenemia pp65 pode ser relevante em pacientes com LES, e estudos com maior número de pacientes são necessários para estabelecer a sensibilidade e a especificidade da antigenemia pp65 em diferentes contextos clínicos envolvendo pacientes com LES.

## INTRODUCTION

Infectious agents are among the main causes of mortality in systemic lupus erythematosus and immunocompromised patients^1^^-^ 3. Human cytomegalovirus (CMV) is a DNA virus, member of the herpesviridae family. A diagnosis of CMV replication and CMV disease can be assessed through various techniques including serology for detection of virus components and histopathologic findings, although the most important laboratory techniques for diagnosis in immunocompromised patients are those that quantify virus molecular components and nucleic acid amplification by polymerase chain reaction (PCR)^4^^,^ 5. For instance, the detection in peripheral blood leucocytes and early monitoring of the phosphoprotein 65 (pp65 antigenemia), abundant detected in viral tegument (or viral matrix) by indirect immunofluorescence, has been effectively used in transplant centers as a means to determine an early therapeutic strategy^6^^-^ 9. However, the effect of its use in patients with autoimmune diseases is still unclear and it has not been extensively explored in scientific literature yet^(5, 10, 11)^.

SLE patients presenting fever are a challenge for physicians, which can be facing a complex problem: fever may be the result of disease activation and can occur without an infection. Besides, a flare can itself be caused by various infectious agents. In those situations, an extensive list of tests are routinely performed. However, laboratory tests for pp65 antigenemia are not always done. This case series describes an active tracking study of pp65 antigenemia conducted in hospitalized SLE patients with suspected and unclear infection. The purpose was to document viral replication of CMV as a possible etiological infection agent.

## MATERIALS AND METHODS

This is an observational and descriptive series of hospitalized SLE cases from March 2015 to March 2016 in the Antonio Pedro University Hospital, Niteroi, and the Pedro Ernesto University Hospital, Rio de Janeiro, Brazil. The criteria for inclusion consisted of hospitalized SLE patients under multi-professional care, independent of age and gender, who have been previously immunosuppressed and who were hospitalized due to a suspected infection. Patients newly diagnosed with SLE and starting an induction immunosuppression therapy for SLE, SLE patients hospitalized for general procedures not primarily related to SLE activity, renal-transplanted SLE patients, as well as patients diagnosed with cancer, HIV, syphilis, viral hepatitis B or C, and pregnant women were excluded from the study. Patients with a promptly identifiable cause of infection, particularly bacterial in origin, for example from skin, urinary tract or pneumonia, as well as positivity in blood cultures were also not considered in this study.

SLE was classified according to the criteria of the *Systemic Lupus International Collaborating Clinics* (SLICC)^12^. The presence of CMV replication was assessed through pp65 antigenemia on cellular samples obtained from peripheral blood. Clinical findings and laboratory test results were obtained from the patients' medical files. During the first physical evaluation of the cases, the SLE activity was estimated by the use of the SLEDAI 2K (Systemic lupus erythematosus disease activity index 2000). To perfom that, we used the same first blood sample collected to assess pp65 antigenemia to obtain also other complementary exams, such as the dosage of C3, C4 e anti-DNA antibody^13^. A general evaluation of morbidity was also performed using SDI score (Systemic Lupus International Collaborating Clinics/American College of Rheumatology Damage Index for Systemic Lupus Erythematosus)^14^. The research was approved by the Research Ethics Committee of the Fluminense Federal University (UFF), number CAEE: 43049215.2.0000.5243.

**Figure 1:**
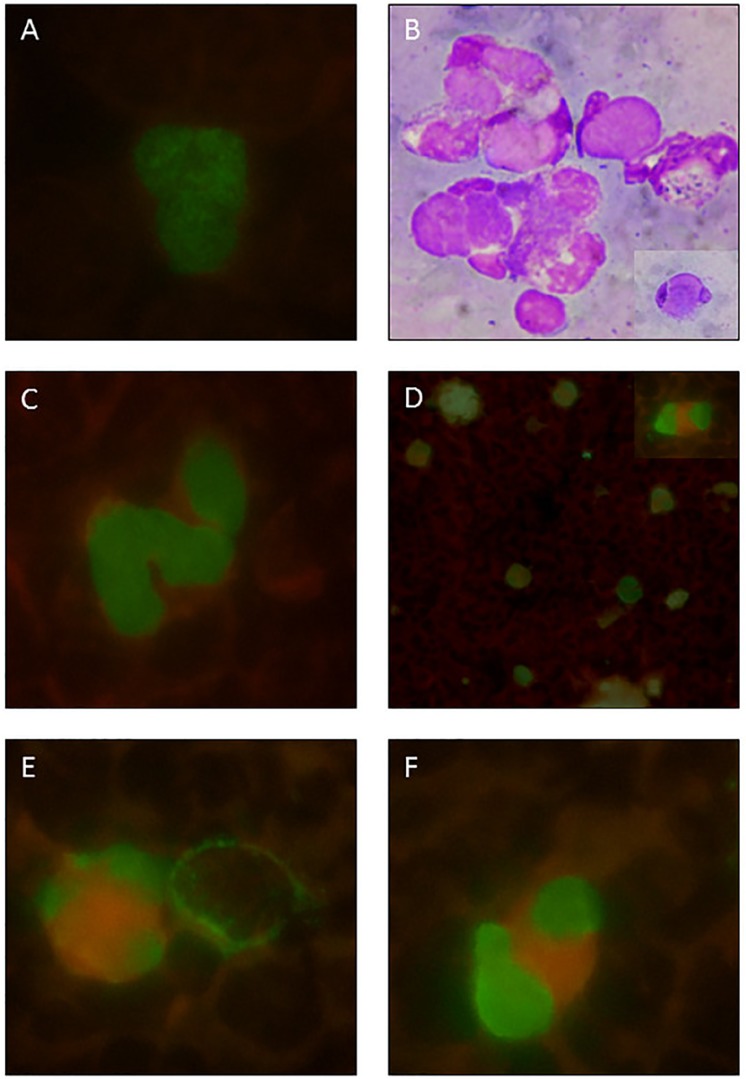
Representative photomicrographs of positive pp65 antigenemia (immunofluorescence). A) pp65 positive neutrophil from a renal transplant patient infected with CMV, used as a reference due to its typical nuclear granular appearance. B) LE cells: neutrophils containing cytoplasmic amorphous inclusions and peripheral nucleus (Wright staining); a detailed view is shown at the lower right corner. C) From case 5: pp65 positive neutrophils with a smooth granular appearance. D) From case 6: panoramic view of pp65 positive neutrophils and cells similar to LE cells; a detailed view is shown at upper right corner. E) and F) From the same SLE case 6: similar dysmorphic aspects of vacuolated cells, not usually seen in transplanted kidney patients.

The assessment of CMV pp65 antigenemia was performed using a commercial immunofluorescence kit, *CMV turbo Brite* (Netherlands). The test uses monoclonal antibodies specific for the pp65, which appears in early stages of the CMV replication, and the results were expressed by the number of positive cells in 2 × 10^5^ leukocytes (Figure 1). The patients with suspected infection and a positive test for pp65 antigenemia had a re-evaluation of pp65 antigenemia after 15 and 30 days. When tested, total DNA was extracted from 200 mL of whole blood using QIAamp DNA mini Kit (Qiagen, Germany), following the manufacturer's protocols. A real-time quantitative PCR assay for CMV DNA was performed using the commercial kit CMV Q-PCR Alert Kit (Nanogen Advanced Diagnostics, Italy) and a 7300 Real-Time termo-cycler (Applied Biosystems, EUA), with the UL123 gene as target region.

## RESULTS

This was a laboratory-based study that included 19 patients presenting with fever, leukopenia/lymphopenia, anti-CMV/IgM positivity or any organ-systemic manifestation suggesting a possible diagnosis for CMV, as an unclear infection. The assessement of pp65 antigenemia was done in a blood sample in parallel to the laboratory routine tests. The results of antigenemia pp65 were presented to the attending physicians, who conducted further independent evaluations and used clinical judgment for diagnosis and CMV treatment options.

There was positivity to pp65 antigenemia in seven patients (36.8%), Table 1. The mean age was 33.5 ± 11.2 years, there were 16 (84%) females, and 16 (84%) were black. A more detailed clinical history of the seven positive patients is described below. Of the seven patients positive for pp65 antigenemia, only three presented positive results for anti-CMV/IgM. Patients who tested positive to pp65 commonly presented lymphopenia, anemia, and higher scores of SLEDAI. Five patients were treated with a specific antiviral therapy (ganciclovir) and, one died from causes attributed to cytomegalic disease (case 4). The CMV-treated patients (ganciclovir, combined or not with intravenous immunoglobulin) were also actively monitored by pp65 antigenemia after 15 and 30 days, with significant progressive disappearance of positivity. Some clinical and laboratory data were compared between patients presenting or not positive pp65 antigenemia confirming the statistical significance to lymphopenia, anemia, and SLEDAI (Table 2).

**Table 1:** Clinical and laboratorial characteristics of the patients that presented pp65 antigenemia positivity

Parameter	Case 1	Case 2	Case 3	Case 4	Case 5	Case 6	Case 7
Age, years	38	9	49	45	18	40	27
Ethicity, skin colour	Brown	Black	White	Brown	White	White	Black
SLICC, criteria number	5	8	6	11	6	4	8
Time of SLE disease, years	7	<1	3	8	5	15	<1
SDI, criteria number	2	0	2	0	0	0	1
SLEDAI 2K, criteria number	25	36	24	21	12	23	17
Hemoglobin, g/dL	7.2	5.8	8.7	7.3	10.2	9.5	6.0
Lymphocytes/mm^3^	352	410	350	330	1100	465	517
ESR, mm/h	59	93	127	120	70	68	80
C Reactive protein, mg/dL	4.8	49.5	0.6	29.1	1.2	1.2	9.6
C3/C4	47/10	40/8	74/16	49/19	17/4	58/12	54/3
Last year immunesupressors	PD/MMF	Zero	PD	PD/MMF	MP/PD/MMF	PD/CsA	Zero
Current immunesupressors	PD	MP/CPM/RTx	CFM	DEX/CsA	PD/MMF	PD	DEX
Anti-CMV/IgM	NEG	POS	NEG	POS	NEG	NEG	POS
pp65, céls/2x10^5^	38	7	5	12	60	162	93
Ganciclovir/IG	GAN	GAN/IG	Não	GAN*	IG	GAN	GAN/IG

Abbreviatons: ESR = Erythrocyte Sedimentation rate: GAN = ganciclovir; IG = endovenous immunglobulins; PD = prednisona; MP = metilprednisolone pulses; CPM = ciclophosphamide; DEX = dexametasone; CsA = cyclosporine; MMF = micophenolate mofetil; RTx = Rituximab.

**Table 2:** Clinical and laboratory baseline characteristics of SLE patients according to pp65 antigenemia positivity.

Parameter	POS (n=7)	NEG (n=12)
Age, years	32.9 ± 14.9	33.8 ± 9.1
Gender, Female	6/7 (85%)	10/12 (83%)
Ethnicity (non-white)	4/7 (57%)	12/12 (100%)
SLEDAI	21.7 ± 8.5	9.0 ± 5.2 *
Hemoglobin, g/dL	7.9 ± 1.8	9.5 ± 2.8 *
Leucocytes /mm^3^	7.029 ± 4.424	8.509 ± 4.332
Neutrophils /mm^3^	5.843 ± 3.811	6.593 ± 3.161
Lymphocytes/mm^3^	503 ± 272	1.089 ± 1.093 *
Eosinophil/mm^3^	123 ± 273	80 ± 106
Platelets /mm^3^	304 ± 124	222 ± 113
ESR, mm/h	89 ± 29	71 ± 43
C reactive protein, mg/dL	13.7 ± 18.7	5.5 ± 8.2
Glycose, mg/dL	128 ± 58	107 ± 55
Urea, mg/dL	38 ± 24	62 ± 39
GFR, mL/min	85.9 ± 41.3	57.4 ± 47.5
Serum albumin, g/dL	2.5 ± 0.5	2.5 ± 0.6
Serum globulins, g/dL	3.7 ± 0.7	3.0 ± 0.7
Cholesterol, mg/dL	193 ± 82	197 ± 65
Triglycerides, mg/dL	269 ± 141	187 ± 78
LDH U/L	378 ± 191	480 ± 371
C3, mg/dL	52 ± 19	72 ± 31
C4, mg/dL	11 ± 7	16 ± 10
Anti-DNA, U/mL	104 ± 120	100 ± 140

Continuous data are reported as mean ± standard deviation and categorical as frequency and percentage (%). Differences between groups were evaluated by Mann-Whitney test or by Fisher's exact test, respectively. A P value was considered significant if <0.05 (*).

To the better understand some atypical appearence on neutrophil immunofluorescence of pp65 antigenemia in SLE patients, we made photos in order to compare SLE cases with standard cases of kidney transplant. Figure 1 shows representative images of pp65 positive cells from our standard experience with kidney transplant cases, and also new findings and conjectures about the dysmorphic pp65 positive cells with similarities in aspect of LE cells found in SLE cases in this series.

### CLINICAL HISTORIES OF THE CASES

Case 1. A 38-year-old woman with SLE for seven years, presented polyarthritis, serositis, proteinuria, and acute renal failure. She was ANA positive and anti-Sm positive. The renal biopsy identified Class III lupus nephritis associated to membranous findings (class V). There was only partial remission following six monthly pulses of metilprednisolone and cyclophosphamide and then switched to maintenance with MMF. The current hospitalization was due to fever, followed by acute mental confusion and worsening of proteinuria. Infection screening included blood and urine cultures, imaging exams, and cerebrospinal fluid puncture, but results were not conclusive. She received vancomycin and ceftriaxone empirically with no clinical improvement and after 3 weeks, a pp65 antigenemia was requested and showed positivity. By this time, a confirmation of CMV by the viral load from whole blood was obtained. Treatment with ganciclovir was started, followed by fever disappearance and clinical and laboratory improvement, including partial reduction of proteinuria.

Case 2. A 9-year-old girl who developed SLE during the previous year characterized by hemolytic anemia, polyarthritis, pleuritis, pericarditis, and proteinuria. She presented positivity to ANA, anti-dsDNA and lupus anticoagulant test as well as complement consumption. The condition evolved into a severe disseminated disease including cardiac valvar lesions, pancreatitis, and renal dysfunction. Dialysis, mechanical ventilation, and several blood transfusions were required. She also presented generalized convulsive crisis followed by hemodynamic instability, and had a long stay in the intensive care unit. She was submitted to several microbiological studies and antibiotic schemes. Besides, she was submitted to different immunosuppressive therapy attempts with corticosteroids pulse therapy, plasmapheresis, cyclophosphamide, intravenous immunoglobulin and rituximab. After an initial clinical improvement and hemodynamic stabilization, she persisted with low-grade fever and leukopenia. At this stage, she had a positivity for anti-CMV/IgM, and a further investigation for pp65 antigenemia was positive. She was treated with ganciclovir for six weeks, until pp65 antigenemia became negative. After a long hospitalization, she had a progressive clinical improvement and hospital discharge.

Case 3. A 49-year-old woman diagnosed with SLE three years before had skin lesions, alopecia, and was ANA positive including positivity to anti-Sm, anti-dsDNA, and complement consumption. Three months before the admission, she developed lupus nephritis with nephrotic range proteinuria, dysmorphic hematuria, and a positive direct Coombs. Nephritis was treated with endovenous corticosteroids and cyclophosphamide. She was admitted due to fever, mental disorientation, and hallucinations. She was empirically treated with antibiotics. Screening for CMV infection showed positive but low pp65 antigenemia, and no specific treatment for CMV was performed. She evolved well.

Case 4. A 45-year-old SLE female patient who had been admitted eight years before for photosensitivity, oral ulcer, polyarthritis, hemiparesis, retinal vasculitis, depression and polyneuropathy associated with lymphocytopenia and hemolytic anemia. She presented positive ANA, anti-Sm, anti-dsDNA, C3 and C4 consumption, and proteinuria. Her initial treatment included prednisone and MMF. After four years, a pulmonary tuberculosis occurred. One year before admission, she presented biopsy-confirmed lupus panniculitis having developed bilateral breast nodules including steatonecrosis with some gross microcalcifications. The current hospitalization was due to fever and dyspnea with a diagnosis of pneumonia, which progressed to sepsis. She was submitted to several blood transfusions. The general clinical status had no significant improvement, when an investigation for CMV using pp65 antigenemia was positive. Specific treatment for CMV with ganciclovir started with a fast initial improvement, including the start of weaning from mechanical ventilation. However, on the tenth day of ganciclovir treatment an unexpected clinical worsening occured, with decreasing consciousness and death. A positive DNA-viral load for CMV was still present.

Case 5. An 18-year-old woman diagnosed with SLE five years before, when she presented malar exanthema, polyarthritis, pleural effusion, and lupus nephritis (IV) with proteinuria of 3.2 g/day (anti-dsDNA positive). She was taking MMF, prednisone, and hydroxychloroquine. Two months before the current hospitalization she was hospitalized for sepsis after a cutaneous trauma on her thigh followed by infection. Blood culture identified *S. pyogenes* and she was treated with antibiotics. However, there was only partial improvement and after three weeks she presented erythematous cutaneous lesions, splenomegaly, diffuse lymph node enlargement, hypertriglyceridemia, and low serum fibrinogen. A diagnosis of macrophage activation syndrome was stablished. A pp65 antigenemia investigation was positive. Initially, the treatment included intravenous immunoglobulin and high doses of prednisone, without having been treated with ganciclovir. After a good clinical response, she was discharged from hospital.

Case 6. A 40-year-old woman with history of SLE for fifteen years, characterized by urticarial vasculitis, polyarthritis, haemolytic anemia, and positive ANA. She had evolved with periods of reactivation and remission of cutaneous and hematological manifestations. Over time, the treatment included hydroxychloroquine, azathioprine, dapsone, methotrexate, and cyclosporine with variable responses, and in addition, low dose of steroids. Current hospitalization was due to fever, low back pain and criteria for urinary sepsis. There was nephrotic range proteinuria and an investigation confirmed left renal vein thrombosis. A diffuse infiltrate was also present in the right lower lobe of the lungs associated with hepatosplenomegaly. Pp65 antigenemia was positive, but no specific antiviral treatment was prescribed. There was initial improvement with antibiotic therapy for urinary infection and the patient was discharged of the intensive care unit. However, because of clinical worsening two weeks later and maintained pp65 antigenemia positivity, ganciclovir was started. There was a significant clinical improvement, and after 15 days, the laboratorial monitoring showed negative pp65 antigenemia.

Case 7. A 27-year-old man was diagnosed with SLE, clinically characterized by pleurisy, arthritis, and non-nephrotic proteinuria associated with positivity for antinuclear antibodies (ANA), anti-double stranded DNA (anti-dsDNA), anti-Sm, and complement consumption. Because a purulent pleural fluid and positivity for adenosine deaminase (ADA) was found, he was treated for tuberculosis. After three months, he developed bacterial endocarditis. A blood culture was positive for coagulase-negative staphylococci. Since then, he has been under several and prolonged antibiotic therapies as well as blood transfusions. He was using oral corticosteroid and presented persistent fever. Pp65 antigenemia was positive and ganciclovir treatment was started. There was fever decrease, but occasional peaks were still observed until the end of the third week on ganciclovir. Gallium scintigraphy showed endocardial uptake and a transesophageal echocardiogram revealed mitral perforation. A new antibiotic approach was carried out. He also used high doses of intravenous immunoglobulins. A right-sided Parsonage-Turner plexopathy, which was attributed to CMV infection, completely improved with the use of ganciclovir. He was referred to cardiac surgery due to valve injury.

## DISCUSSION

This study is the result of a retrospective assessment of hospitalized SLE patients that presented fever and clinical findings consistent with an unclear infection. The relationship between CMV infection and SLE are not well characterized yet. The risk factors for CMV, the active disease and its clinical manifestation and treatments for SLE are still not well defined. Primary cytomegalovirus infection is usually asymptomatic in immunocompetent people, but may manifest as CMV mononucleosis in about 10% of adults, characterized by fever, liver dysfunction, and lymphocytosis with a usually mild self-limited course. However, severe CMV disease can occur in immunosuppressed individuals. SLE is one of the diseases that best exemplifies autoimmune diseases. Therefore, we investigated the frequency of a CMV replication biomarker (pp65 antigenemia) in a group of hospitalized SLE patients.

CMV is an important infectious agent not always ruled out in a systematic way. Infections remain one of the main causes of morbidity and mortality in SLE patients. In clinical practice, despite of the ubiquitous presence of this pathogen in general population, it seems that a diagnosis of CMV infection is not made as frequently as expected. The reasons for that are unclear, but it is likely to occur due to a lack of availability of highly accurate diagnostic methods, even in referral centers^15^. Moreover, on one hand, some clinical features of CMV infection remind clinical findings of SLE flare, and on other hand, lupus patients are prone to viral infection or reactivation of latent viruses due to the lupus itself and/or because of the immunosuppressesive therapy. Large population studies are needed to asses all these possibilities.

Despite the high mortality rate of the more invasive forms^16^, the impact of CMV infections on the morbidity and mortality of SLE patients is still poorly documented. However, in clinical practice, CMV has been associated with vasculopathies, Raynaud's phenomenon^17^, fevers of undetermined origin, pneumonia, myocarditis, nephritis, meningoencephalitis as well as the appearance of antiphospholipid antibodies^18^. Invasive forms have been documented mainly through biopsies (or necropsies) in which the goal was the detection of inclusion bodies or immunohistochemistry markers in tissue^16^. In general, in transplantation medicine, pp65 antigenemia is less sensitive in relation to RT-PCR, especially in invasive gastrointestinal disease or in the presence of neutropenia. However, in invasive gastrointestinal tract disease, whether in organ transplants or in inflammatory diseases such as Crohn's or ulcerative colitis, the use of tissue biopsy study including immunohistochemistry with direct detection of CMV is of great value^18^. In general, CMV infection is very difficult to determine only by clinical data. For example, in our findings, the serologic studies (anti-CMV/IgG or anti-CMV/IgM) were not reliable for indicating or ruling out a possible primary infection or disease reactivation. Therefore, based on clinical data suggestive of CMV disease, the absolute majority of medical centers have used the availability of molecular assays like PCR to make the diagnosis of CMV^4^^,^ 5. Questions about the possibility of SLE patients of producing antibodies under pathological conditions, and if that could compromise the CMV diagnosis by serology, or even the pp65 antigenemia itself, are still unanswered.

CMV viremia is very prevalent among immunosuppressed patients, and various diagnostic strategies have been commonly used in clinical practice, for example PCR and pp65 antigenemia. Some transplantation centers monitor antigenemia closely, and to prevent CMV complications phisicians start promptly preemptive and /or prophylactic treatment, mostly in the first six months after transplantation^31^. However, data about CMV monitoring in autoimmune diseases are scarce, as well as protocols about when and how to monitor CMV infection in these autoimmune patients. In this study, we were interested in applying the immunofluorescence technique with monoclonal antibodies that are attached to the phosphoprotein 65 in circulating leucocytes, as an indication of active CMV replication. This technique, first described in 1988^19^, has been proven effective and faster than viral isolation. Therefore, it is largely applied to monitor patients who undergo organ transplant^20^. As is the case of our center, the availability of well-trained staff for CMV pp65 antigenemia detection tests is important to reduce the degree of subjectivity in the interpretation of tests. In our study, we have considered as positive any pp65 nuclear immunostaining present in slides, irrespective of the cell aspect, and despite the presence of subtle granular differences or vacuolar changes (Figure 1), which deserves future attention.

As mentioned earlier, CMV infection in patients with lupus can trigger or even worsen disease activity, which is associated with a higher mortality rate. On the other hand, inflammation is one of the mechanisms that can reactivate latent CMV^18^. SLE is characterized by periods of acute disease and remission. Patients with autoimmune diseases, including those presenting positive blood cultures for bacteria and/or fungi, can also be co-infected with CMV^10^. Some studies have suggested a possible link between the CMV and SLE activity^5^^,^ 21 ^,^ 22, and CMV infection has been gaining attention as a potential complicating factor in the SLE immunosuppressive context^10^^,^ 23. Although the occurrence of CMV infection in SLE is well described, the role of the virus as the etiological factor of SLE is not well established, and the association of lupus with CMV seroprevalence is unclear^24^. The chronic inflammation associated with autoimmunity promotes an ideal microenvironment where a latent CMV can be reactivated. Processes involving T cell activation and inflammation tend to facilitate this reactivation^18^.

The case series presented in this study showed that it is possible to make an early decision and even treat CMV infections using very effective and specific antiviral therapy^25^. However, the relationship between CMV infection and SLE activity, or whether CMV could have been reactivated due to intense inflammation and sepsis in some cases is not completely clear. In addition, whether the inflammatory improvement could also determine a reduction of viral replication is indefinite^18^. Our cases must not be considered as having cytomegalic disease, but only suspicious CMV cases, as tracking of pp65 antigenemia was conducted. However, in the majority of our cases, there was significant clinical improvement after the introduction of specific antiviral therapy. For example, multiple indirect antiviral effects might occur from intravenous immunoglobulin, which would be a therapeutic option for macrophage activation syndrome, lupus activity, and even CMV disease itself^26^. Similarly, specific co-infections might be present, considering a case where a recently cured tuberculosis was correlated to lupus activity as well as a compromised neural plexus^27^^,^ 28.

The pp65 antigenemia is a rapid test, with results available within 3-5 hours after sampling,^29^^),^ much faster than the PCR assay. A small sample of peripheral blood is needed, and the test has a relatively low cost (compared with PCR) and high specificity^30^. The test allows repeating the CMV replication as many times as needed throughout the clinical investigation and monitoring treatment results through variations in viral replication, which is comparable to the tracking used in organ transplanted patients^31^. On the other hand, the validity of pp65 antigenemia as a diagnostic tool for patients with SLE presents some aspects that need to be discussed further. For instance, it is known that the cutoff value for renal transplant recipients varies from 8 to 20 cells/200,000 leukocytes (92% sensitivity and 70% specificity) depending on the center^32^. In bone marrow transplanted patients, a single positive cell is enough to be considered as a positive result^33^. Therefore, routine CMV monitoring in transplantation can be used as a preemptive approach, so that pp65 antigenemia might work as a marker of infection or viral replication, as performed in our group^31^. In 2013, a study^10^ reported a *cutoff* value of 10 cells per 200,000 leukocytes to associate CMV with mortality in autoimmune diseases ( 75% sensitivity and 72.2% specificity).

SLE patients with lymphopenia must be closely monitored for CMV, due to the high risk associated with high CMV viral load^11^. We observed in our clinical histories high rates of blood transfusions, which is of concern and should be assessed as one of the routes of CMV transmission. New infections by new strains can occur, usually leading to a new primary infection, particularly serious in immunosuppressed patients. That means that patinents could face a primary infection, a reactivation or even a new infection by a different CMV strain, causing a constant worry: infection or disease activity versus infection and diseases activity. A strategy to prevent this route of transmission, other than transfusion of CMV seronegative components, is the filtering of blood components in order to retain leukocytes^34^^,^ 35.

In conclusion, among patients with unclear causes of infection, pp65 antigenemia positivity may be significantly frequent. From our study, it was not possible to state whether the detected CMV replication was related to cytomegalic disease and whether the antiviral medication benefited the patients. Nevertheless, a simple and cheap pp65 antigenemia test, associated with suggestive clinical and laboratory findings, could contribute to an early decision regarding antiviral treatment. More studies with a greater number of patients are needed to clarify matters such as sensitivity and specificity in different clinical contexts of SLE disease.
